# Very Early-Onset Inflammatory Bowel Disease (VEO-IBD) Presenting with Recurrent Leukocytoclastic Vasculitis Preceded by Streptococcal Pharyngitis

**DOI:** 10.1155/2021/1996430

**Published:** 2021-05-20

**Authors:** Ashley Fonseca, Julee Sunny, Lina M. Felipez

**Affiliations:** ^1^Department of Medical Education, Nicklaus Children's Hospital, Miami, FL, USA; ^2^Department of Pediatric Gastroenterology, Hepatology, and Nutrition, Nicklaus Children's Hospital, Miami, FL, USA

## Abstract

Inflammatory bowel disease (IBD) that presents in children <6 years of age is known as very early-onset IBD (VEO-IBD). Extraintestinal manifestations in IBD, such as erythema nodosum (EN), pyoderma gangrenosum (PG), and, less likely, leukocytoclastic vasculitis (LV), are more commonly present in Crohn's disease. Association between LV and ulcerative colitis (UC) is not commonly seen. We report a case of a 6-year-old female with a VEO-IBD UC phenotype presenting with multiple episodes of leukocytoclastic vasculitis, each preceded by streptococcal pharyngitis. Prior to the diagnosis of VEO-IBD, a skin biopsy was obtained and had shown leukocytoclastic vasculitis with a negative IgA stain. Initial laboratory results were remarkable for leukocytosis and increased anti-strep O and anti-DNase B titers. Gastrointestinal panel PCR demonstrated *Clostridium difficile* toxin A/B. Treatment for LV consisted of methylprednisolone IV 20 mg for four days with a weaning schedule of prednisolone for two weeks and naproxen 250 mg BID for three days. *Clostridium difficile* was treated with metronidazole 250 mg TID for ten days. She remained stable for three years until she presented with continuous bloody stools, newly onset chest pain, and shortness of breath. Computed tomography angiogram (CTA) was normal. Stool calprotectin was elevated at 658 mcg/gm. Abdominal magnetic resonance enterography (MRE), esophagogastroduodenoscopy, and colonoscopy confirmed a VEO-IBD ulcerative colitis phenotype. She was started on infliximab 10 mg/kg every four weeks after infliximab titers, and antibodies were obtained. Currently, the patient remains on clinical and biochemical remission, with no recent LV episodes or recurrence of streptococcal pharyngitis. Our patient is unique as no case report has been published with multiple episodes of leukocytoclastic vasculitis in association with a VEO-IBD UC phenotype.

## 1. Introduction

Inflammatory bowel disease (IBD) that presents in children <6 years of age is known as very early-onset IBD (VEO-IBD). VEO-IBD is a heterogeneous disease that, in some cases, can be severe and refractory to treatment in comparison with older children and adults. Given its early presentation, VEO-IBD may present as a monogenic disease associated with primary immunodeficiencies in up to 15–20% of the patients with VEO-IBD [1]. History of infant-onset disease, perianal disease, infection history, and association with other autoimmune disorders may prompt the clinician to evaluate for VEO-IBD. A detailed clinical history, evaluation of humoral immunity and antibody deficiency, and genetic testing, such as next-generation sequencing (NGS), are crucial elements in diagnosing VEO-IBD monogenic disease. More than 50 genes have been associated with VEO-IBD and have helped clinicians deliver targeted therapy [1].

IBD can often be associated with extraintestinal manifestations (EIMs), including cutaneous, joint, and ocular problems. These EIMs are more frequently observed in Crohn's disease (CD) than in ulcerative colitis (UC) [2]. The most common cutaneous manifestations of IBD are erythema nodosum (EN) and pyoderma gangrenosum (PG). While psoriasis, Sweet's syndrome, dermatitis herpetiformis, epidermolysis bullosa acquisita, necrotizing vasculitis, and leukocytoclastic vasculitis (LV) are more rarely observed [2]. Cutaneous manifestations tend to proceed intestinal presentations. We report the unique case of a 6-year-old female with a VEO-IBD UC phenotype who presented with multiple episodes of leukocytoclastic vasculitis, each preceded by streptococcal pharyngitis.

## 2. Case Report

A previously healthy 5-year-old Hispanic female initially presented with intermittent abdominal pain, fever, bloody diarrhea, and emesis for five consecutive days. Laboratory results were remarkable for stool positive *Adenovirus*, *Helicobacter pylori* Ag, and *Cryptosporidium* infections. Due to an infectious etiology, endoscopy/colonoscopy was not performed. She was treated with supportive care, nitazoxanide 200 mg BID for three days, and triple therapy with amoxicillin 50 mg/kg/day BID and clarithromycin 20 mg/kg/day BID for fourteen days, and pantoprazole 20 mg BID for thirty days. Five months later, she presented to an outside hospital with rhinorrhea, diarrhea, and pain and swelling of her distal lower extremities with an associated petechial rash of the palms, soles, and legs. A rapid strep test was positive and was treated with IM penicillin. During the following months, the patient had six similar episodes afterward, each preceded by streptococcal pharyngitis and treated with antibiotics. One month prior to presentation to our hospital, she presented with a severe episode to an outside hospital and underwent a skin biopsy that showed leukocytoclastic vasculitis with a negative IgA stain. Rheumatology started her on treatment with colchicine for these recurrent episodes.

At six years of age, she again presented to our institution with bilateral swelling of the hands and lower extremities, an inability to bear weight, a petechial rash of the palms, soles, and legs, and diarrhea with specks of blood. During her initial presentation, workup was remarkable for a leukocytosis of 16.0 10 K/uL and increased anti-strep O and anti-DNase B titers of 1,800 IU/mL and 428 U/mL, respectively. No signs of anemia, proteinuria, or hematuria were seen. Gastrointestinal panel PCR was remarkable for the detection of *Clostridium difficile* toxin A/B (Table 1). Rheumatoid factor, serum IgA, serum IgM, and C3 and C4 complement were normal. Serum IgG levels were markedly elevated (1,436 mg/dL). Perinuclear antineutrophil cytoplasmic antibodies (p-ANCA) were not detected. Vaccine titers were remarkable for low levels of *Haemophilus influenzae B* IgG antibody titers. Lymphocyte subset analysis demonstrated mildly low levels of T-cells (Table 2). Treatment for LV consisted of methylprednisolone IV 20 mg for four days with a weaning schedule of prednisolone for two weeks and naproxen 250 mg BID for three days. *Clostridium difficile* was treated with metronidazole 250 mg TID for ten days.

**Figure 1:**
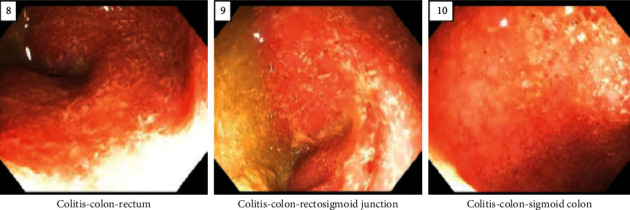
Colonoscopy showing colitis at the rectum, rectosigmoid junction, and sigmoid colon.

She remained stable for three years with no follow-up until she presented with anterior chest pain with radiation to the left shoulder, shortness of breath, and continuous bloody stools. Computed tomography angiogram (CTA) was normal. Stool calprotectin was elevated at 658 mcg/gm. Abdominal magnetic resonance enterography (MRE) revealed thickening of the transverse colon, distal colon, sigmoid colon, and rectal wall, with chronic inflammation noted in the sigmoid colon and distal colon. The small bowel was unremarkable. Esophagogastroduodenoscopy was significant for mild chronic gastritis in the antrum and body of the stomach. Colonoscopy was performed up to the splenic flexure due to increased mucosal friability and patient safety. Pathology was remarkable for moderate to severe chronic active colitis at the distal colon, sigmoid colon, and rectum (Figure 1). An Early-Onset Inflammatory Bowel Disease Panel including sequencing and NGS-based copy number variation analysis for monogenic disease did not detect any pathogenic variant. A preliminary diagnosis of VEO-IBD ulcerative colitis phenotype was made. She was started on infliximab (IFX) 10 mg/kg induction. Initial Anser IFX at week 8 showed a level of 12.4 ug/mL and antibodies to infliximab (ATI) of <3.1 unit/mL. Infliximab frequency was decreased to every four weeks to achieve therapeutic levels. Currently, the patient remains on clinical and biochemical remission, with no recent LV episodes or recurrence of streptococcal pharyngitis. Repeat esophagogastroduodenoscopy and colonoscopy are recommended to further classify the patient's phenotype and assess mucosal healing.

## 3. Discussion

Among the cases of VEO-IBD, monogenic VEO-IBD with its associated primary immunodeficiencies represents approximately 15–20% of VEO-IBD patients. Patients that present very young and with a history of folliculitis, dermatitis, significant infections, and other autoimmune diseases are more strongly associated with a monogenic VEO-IBD [1]. A detailed clinical history, evaluation of humoral immunity and antibody deficiency, and genetic testing, such as next-generation sequencing (NGS), are crucial elements in diagnosing VEO-IBD monogenic disease. Immunological workup, including immunoglobulin levels, vaccine titers, and lymphocyte subset analysis, evaluates humoral immunity and cytotoxic B and T-cell immunity and can steer physicians towards a monogenic VEO-IBD diagnosis [1]. Additionally, genetic testing, such as NGS, is a vital tool for diagnosing monogenic VEO-IBD [1]. Since our patient initially presented with recurrent infections and episodes of bloody diarrhea, an associated primary immunodeficiency was considered. The mildly low T-cell levels and *H. influenzae B* titers were not suggestive of a primary deficiency (Table 2). Also, an Early-Onset Inflammatory Bowel Disease Panel including sequencing and NGS-based copy number variation analysis for monogenic disease did not detect any pathogenic variant.

Leukocytoclastic vasculitis is characterized by inflammation of the postcapillary venules with neutrophilic infiltration and nuclear debris [4]. LV can be triggered by multiple drugs, infections, malignancies, and systemic and autoimmune disorders [5]. The literature describes that a rash can present in the lower extremities (83%), upper extremities (42%), buttocks (25%), and trunk (25%) [2]. As demonstrated by Sais et al. (1998), leukocytoclastic vasculitis may also have different immunological laboratory characteristics. In a population of 160 patients with leukocytoclastic vasculitis, they found some of the following results: rheumatoid factor (≥40 U/mL) (26.4%), p-ANCA (21.0%), increased serum IgA levels (24%), and decreased C3 plasma levels (6%) and C4 plasma levels (25%) [6]. However, our patient had no abnormality in the aforementioned laboratory levels (Tables 1 and 2). Given LV affects small vessels, it is imperative to rule out a systemic disease with possible renal, joint, gastrointestinal, pulmonary, and neurologic involvement [4]. Fortunately, our patient had no systemic involvement as evidenced by the normal hemoglobin, sed rate, urinary protein/creatinine ratio, urinary blood and RBCs, and rheumatoid factor (Tables 1 and 2).

LV is a rare extraintestinal manifestation for IBD [3]. One proposed mechanism for LV describes the formation of immune complexes after the inflamed colonic mucosa in UC allows fecal antigen exposure to the submucosal lymphoid tissue [7]. Such immune complexes lead to erythrocyte extravasation and tissue necrosis of the small dermal vessels after complement activation, leukotaxis, the release of lysosomal enzymes, and destruction of the vascular wall [7]. Another hypothesis postulates that IBD acts as a systemic disorder involving different tissues and may act at varying time intervals [3].

Treatment of LV in association with UC includes steroids, mesalamine, dapsone, colchicine, and infliximab, with no proven superiority among them [2].

Our patient is unique as no case report has been published with recurrent episodes of leukocytoclastic vasculitis in association with a VEO-IBD UC phenotype. Repeat esophagogastroduodenoscopy and colonoscopy are recommended to further characterize this patient's phenotype and assess mucosal healing. Fortunately, after appropriate treatment was started, our patient remains on clinical and biochemical remission, with no recent LV episodes or recurrence of streptococcal pharyngitis.

## Figures and Tables

**Table 1:** Pertinent basic and infectious laboratory results of our patient.

Parameter	Result	Normal range for age
White blood cells (WBCs)	16.0	4.8–10.8 10 K/uL
Hemoglobin	12.1	10.6–15.2 gm/dL
Sed rate	14.0	0–30 mm/hr
Urinary blood	Negative	Negative
Urinary red blood cells (RBCs)	1/high-power field	0/high-power field
Urinary protein/creatinine ratio	0.04	0–0.2 mg/mg
Antistreptolysin O (ASO) titer	1,800	0–640 IU/mL
Anti-DNase B	428	0–375 U/mL
Gastrointestinal panel PCR	Detected *C. difficile* toxin A/B	Negative

**Table 2:** Pertinent rheumatological and immunological laboratory results of our patient.

Parameter	Result	Normal range for age
Rheumatoid factor	8.6	0–11.9 IU/mL
Serum IgA	110.7	33.0–200.0 mg/dL
Serum IgG	1,436	608–1,229 mg/dL
Serum IgM	109.8	46.0–197.0 mg/dL
C3 complement	132.0	92.0–161.0 mg/dL
C4 complement	31.5	16.0–42.0 mg/dL
Perinuclear antineutrophil cytoplasmic antibodies (p-ANCA)	Not detected	Not detected
Diphtheria toxoid Ab IgG	0.41	>0.1 IU/mL
Tetanus toxoid Ab IgG	0.26	>0.15 IU/mL
Haemophilus influenzae B IgG Ab	0.21	≥1.0 mg/L
CD3+ T-cells	40.7	53–83%
CD19+ B-cells	44.3	5–21%
CD4+/CD3+ T-cells	27.1	35–51%
CD8+/CD3+ T-cells	12.0	12–37%
CD16+CD56+/CD3-natural killer	12.9	1–11%

## Data Availability

The necessary data needed to support this article are included within the case report.
